# The burden of major adverse cardiac events in patients with coronary artery disease

**DOI:** 10.1186/s12872-016-0436-7

**Published:** 2017-01-04

**Authors:** I-Ting Tsai, Chao-Ping Wang, Yung-Chuan Lu, Wei-Chin Hung, Cheng-Ching Wu, Li-Fen Lu, Fu-Mei Chung, Chia-Chang Hsu, Yau-Jiunn Lee, Teng-Hung Yu

**Affiliations:** 1 Department of Emergency, E-Da Hospital, I-Shou University, Kaohsiung, 82445 Taiwan; 2 Division of Cardiology, E-Da Hospital, I-Shou University, No. 1, Yi-Da Rd, Jiau-Shu Village, Yan-Chao District, Kaohsiung, 82445 Taiwan; 3 Division of Endocrinology and Metabolism, E-Da Hospital, I-Shou University, Kaohsiung, 82445 Taiwan; 4 Division of Gastroenterology and Hepatology, Department of Internal Medicine, E-Da Hospital, I-Shou University, Kaohsiung, 82445 Taiwan; 5 Division of Cardiac Surgery, Department of Surgery, E-Da Hospital, I-Shou University, Kaohsiung, 82445 Taiwan; 6 Lee’s Endocrinology Clinic, Pingtung, 90000 Taiwan; 7 School of Medicine for International Students, E-Da Hospital, I-Shou University, Kaohsiung, 82445 Taiwan; 8 Department of Nursing, I-Shou University, Kaohsiung, 82445 Taiwan; 9 Department of Biomedical Engineering, National Cheng Kung University, Tainan, 70101 Taiwan

**Keywords:** Major adverse cardiovascular events, Predictive factors, Coronary artery disease

## Abstract

**Background:**

Patients with a history of cardiovascular disease are at high risk of developing secondary major adverse cardiac events (MACE). This study aimed to identify independent predictors of MACE after hospital admission which could be used to identify of high-risk patients who may benefit from preventive strategies.

**Methods:**

This study included 1,520 consecutive patients with coronary artery disease (CAD) (654 with acute coronary syndrome (ACS) and 866 with elective percutaneous coronary intervention (PCI) patients) who received PCI and/or stenting. MACE was defined as all-cause mortality or rehospitalization for a cardiovascular- related illness. Cardiovascular-related illnesses included heart failure, reinfarction (nonfatal), recurrence of angina pectoris and repeat PCI or coronary artery bypass graft.

**Results:**

During a mean follow-up period of 32 months, 558 of the 1,520 patients developed at least one MACE. Cox regression analysis showed that the baseline clinical and biochemical variables which associated with MACE were age, being illiterate, a widow or widower, and/or economically dependent, having triple vessel disease, stent implantation, anemia, and/or diabetes mellitus, waist to hip ratio (WHR), diastolic blood pressure, fasting glucose, total cholesterol, high-density lipoprotein cholesterol (HDL-C), creatinine, estimated glomerular filtration rate (eGFR), red blood cell count, hemoglobin, hematocrit, and mean corpuscular-hemoglobin concentration (MCHC) in ACS patients, and age, malnourished, and/or economically dependent, taking hypoglycemic medication, having triple vessel disease, stent implantation, anemia, diabetes mellitus, and/or hypertension, WHR, fasting glucose, HDL-C, uric acid, creatinine, eGFR, high-sensitivity C-reactive protein, mean corpuscular volume, and MCHC in elective PCI patients. Using multivariate Cox regression analysis, we found the MACE’s independent factors are triple vessel disease, stent implantation, hypertension, and eGFR in ACS patients, and having triple vessel disease, stent implantation, hypertension, and uric acid in elective PCI patients.

**Conclusions:**

Having triple vessel disease, stent implantation, hypertension, and eGFR or uric acid independently predicted MACE in patients with CAD after long-term follow-up. Fortunately, these factors are modifiable and should be identified and monitored early.

## Background

Coronary artery disease (CAD) is a major health problem worldwide including Taiwan, and it is expected to be the leading cause of death by 2020 [1–3]. With advances in both coronary artery bypass graft (CABG) surgery and percutaneous coronary intervention (PCI), mortality from CAD has significantly decreased [4]. Moreover, PCI or PCI with stenting and CABG were associated with a long-term safety profile and improving patient outcomes [5, 6]. However, the poor prognosis and high cost of medical care for patients with CAD impose a considerable burden on both their families and society. Therefore, identifying risk factors that affect the prognosis of patients undergoing revascularization is necessary.

Major adverse cardiac events (MACE) are important causes of morbidity and mortality in CAD patients undergoing PCI. The detection and treatment of the risk factors for MACE are critical to improve health and longevity. As expected, the traditional risk factors (e.g. age, sex, total cholesterol, low-density lipoprotein (LDL) cholesterol, high-density lipoprotein (HDL) cholesterol, systolic blood pressure, and smoking) [7] for CAD are associated with disease progression. Using Intravascular ultrasound monitoring of changes in atheroma burden in 3,473 patients, Chhatriwalla et al. demonstrated that the patients with a very low LDL level (≤70 mg/dL) and normal systolic blood pressure (SBP) (≤120 mmHg) had the slowest CAD progression [8]. In addition, in patients with LDL levels ≤70 mg/dL, progression of native coronary atherosclerotic disease was associated with the presence of residual risk factors including high baseline glucose levels, increased level of triglycerides, and small decrease in apolipoprotein B [9]. Previous study also reported that baseline diabetes was the strongest significant cardiovascular disease-related factor for disease progression [10]. Therefore, the purpose of this study was to evaluate whether baseline risk factors such as demographic and clinical characteristics, lipid levels, inflammation, triple vessel disease, stent implantation, anemia, diabetes mellitus (DM), hypertension, and chronic kidney disease (CKD) were associated with the development of MACE in patients with CAD receiving PCI. The results could be important in increasing physicians’ awareness on the importance of regular baseline risk factors screening when caring for patients with CAD.

## Methods

### Study Subjects

**Fig. 1:**
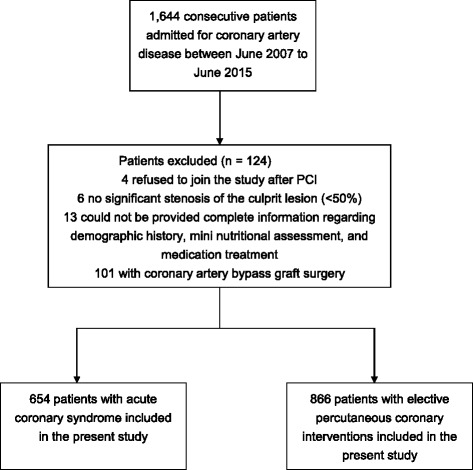
Study flowchart

In this prospective study, from June 2007 to June 2015, we studied 1,644 patients with CAD who made consecutive visits to the Taiwan I-Shout University E-Da Hospital’s Emergency Room and Cardiovascular Clinic (Fig. 1). All the demographic data, clinical information, and current medications of the patients were examined and recorded by two healthcare professionals with expertise in CAD care, via a web-based case report form in the clinical data management system of the E-Da Hospital, with special attention being paid to cardiovascular risk factors and co-morbidities. The clinical data system at the E-Da Hospital documented all the clinical consecutive variables and values when patient admitted to the Cardiovascular Clinic or Ward due to CAD or other heart diseases. Age, sex, smoking habits, hyperlipidemia, arterial hypertension, DM, CAD, history of stroke and acute myocardial infarction were evaluated. Medical history survey, physical examinations, blood test, and urinalysis were conducted. Patients presenting with stage 5 CKD, concomitant inflammatory diseases such as infection or sepsis, and other medical history of malignancy, liver disease or collagen disease, those using steroids, and those who had undergone any type of surgery in the month prior to admission or refused to participate in the study were excluded. Data were evaluated on the day of patients discharge after interobserver agreement gained. In cases of discrepancy, reevaluation by both investigators and a consensus was performed. To detect undiagnosed diabetes, fasting blood glucose concentration was determined at admission. During the hospital stay, repeat blood pressure measurements were conducted to discover undiagnosed hypertension. Written informed consent was given by each CAD patient before enrollment. If patients were unable to give consent because of disease severity, informed consent was obtained from a relative or legal representative. The study protocol and informed consent procedure were approved by the Ethics Committee of I-Shou University E-Da Hospital.

### Laboratory Measurements

Plasma biochemical parameters were measured after an overnight fast. Levels of plasma triglycerides, total cholesterol, LDL-cholesterol, HDL-cholesterol, uric acid, creatinine, and glucose were determined by standard commercial methods using a parallel-multichannel analyzer (Hitachi 7170A, Tokyo, Japan), as described previously [11]. Levels of plasma C-reactive protein (CRP) were measured using a high-sensitivity method (IMMAGE; Beckman Coulter, Immunochemistry Systems, Brea, CA) with a detection limit of 0.2 mg/L. The intra-assay coefficient of variation was 4.2% to 8.7% for high-sensitivity CRP (hs-CRP). Samples were measured in duplicate in a single experiment. Estimated glomerular filtration rate (eGFR) was calculated using the CKD-EPI two-level race equation [12]. Anthropometric parameters including body mass index (BMI) and waist to hip ratio (WHR) were measured. Waist and hip circumferences were measured to the nearest 0.1 cm at the narrowest point between the lowest rib and the uppermost lateral border of the right iliac crest. Hips were measured at their widest point. Seated blood pressure was measured by a trained nurse with a digital automatic blood pressure monitor (model HEM-907; Omron, Omron, Japan) after the subjects had rested for 5 minutes.

### Study End-points

The patients were followed-up after admission using a standardized protocol that included outpatient visits, telephone contact and the recording of recurrent cardiac events. The endpoints were the composite occurrence of MACE, including all-cause mortality or re-hospitalization for a cardiovascular-related illness. Cardiovascular-related illnesses included heart failure, reinfarction (nonfatal), recurrence of angina pectoris and repeat PCI or CABG [13]. The heart failure endpoint was investigated on the basis of clinical parameters suggestive of pulmonary congestion and/or signs of low cardiac output. Angina was definitions of dull diffuse substernal chest discomfort precipitated by exertion or emotion and relieved by rest or nitroglycerin. Non-fatal reinfarction was defined as angina or anginal equivalent accompanied by a new ST-segment elevation in leads consistent with those of the territory of the artery affected in the index event. Revascularization was characterized by the need for percutaneous or surgical intervention motivated by instability of the clinical picture (elective revascularization were not considered endpoints).

### Follow-up

The patients were clinically reevaluated at 3, 6, and 12 months after hospital discharge and then annually until June 2015. All participants had entered a disease management program at our Cardiovascular Clinic. All of the patients received a follow-up questionnaire from a trained nurse during June 2015 to re-evaluate the occurrence of MACE during the whole follow-up period. For the patients did not respond to the questionnaire, personal telephone contact was made. In addition, all hospital discharge reports relating to any other re-admission during the follow-up period were also been reviewed. The performance of PCI and CABG was validated by reviewing the original procedure protocols. Outcomes were assessed by two research physicians with expertise in Cardiology; they have 11 years clinical experience in catheriterization procedure and were active member of the Taiwan Society of Cardiology. Two research physicians were blinded to the patients’ all baseline clinical data. In case of disagreement, consensus was sought. Finally, a cardiovascular interventionist, whose judgment was considered decisive, reviewed all events. In addition, the type of objective information included medical records, radiography, reports of cardiac catheterization, EKG, and cardiac echo, and pathology that were provided the adjudicators to make their assessments according to the endpoint definitions.

### Definitions

In our study, the diagnosis of CAD was confirmed by coronary angiography. Significant coronary stenosis was defined as a vessel luminal diameter decrease of greater than or equal to 70% in either one of 15 coronary segments. If PCI was considered, balloon angioplasty or stenting was performed according the vessel condition. However, for left main and severe triple vessel disease, CABG was suggested to the patients. Hypertension was defined as systolic blood pressure of ≥140 mmHg, diastolic blood pressure of ≥90 mmHg or both, or the use of anti-hypertensive drugs. Hyperlipidemia was defined as a triglyceride level ≥150 mg/dL, and/or a HDL-cholesterol level of <35 mg/dL for men and <39 mg/dL for women, and/or a total cholesterol level of ≥200 mg/dL, and/or a LDL-cholesterol level of ≥130 mg/dL, or those undergoing treatment for lipid disorders according to the ATP III criteria [14]. DM was defined as a glucose level >126 mg/dl [15], or having received treatment for DM. Malnutrition indicator score was defined as normal nutritional status 24 to 30 points, at risk of malnutrition 17 to 23.5 points, and malnourished less than 17 points using the Mini Nutritional Assessment. Patients’ smoking status was classified as never having smoked, former smoker (ceased smoking for at least 1 year), or current smoker. In this study, former and current smokers were analyzed as a group and compared with those who had never smoked.

### Statistical Analysis

Continuous variables were presented as mean ± SD, and categorical variables as a percentage of the total. All statistical analyses were performed using SAS software version 10.0 (SAS Institute, Cary, NC). Baseline characteristics of the patients with MACE and without MACE were compared using the Student’s *t* test or *χ* ^2^ test. Cox regression univariate analysis was performed. The outcome was defined as the time elapsed until the development of the MACE, and all the patients were followed until they developed a MACE. Hazard ratios (HRs) with 95% confidence intervals were estimated by Cox proportional hazards model. The variables with a *p* value less than 0.25 in the univariate Cox regression analysis were entered into the multivariate model. The proportional hazards assumption was further verified by Schoenfeld residuals analysis and assessment of the survival function versus the survival time graph. Model discrimination was assessed by area under the receiver operating characteristic (ROC) curve (AUC) analysis, which is a measure of overall predictive discrimination. The AUC is an overall summary of diagnostic accuracy. AUC equals 0.5 when the ROC curve corresponds to random chance and 1.0 for perfect accuracy. On rare occasions, the estimated AUC is <0.5, indicating that the test does worse than chance [16]. All of the statistical analyses were two-sided, and a *p* value less than 0.05 was considered to be significant.

## Results

Among 1,644 consecutive CAD patients, 124 were excluded due to the following reasons: four patients refused to join the study after PCI, six patients had no significant stenosis of the culprit lesion (<50%), one hundred and one with coronary artery bypass graft surgery, and 13 patients could not be provided complete information regarding demographic history, mini nutritional assessment, and medication treatment. The final study population included 1,520 patients (654 with ACS and 866 with elective PCI patients; Fig. 1). Of the 1,520 patients, 1,107 (72.8%) men and 413 (27.2%) women, mean age of patients is 69 ± 12 years (range, 30 to 99 years). 582 (38.3%) had DM, 949 (62.4%) had hyperlipidemia, and 1,092 (71.8%) had hypertension (Tables 1 and 2).

**Table 1:** Baseline characteristics and clinical data of the study participants with and without major adverse cardiovascular events

Variable	Total *n* (%)	With MACE *n* (%)	Without MACE *n* (%)	*P* value
No	1520	558 (36.7)	962 (63.3)	
Gender				
Women	413 (27.2)	140 (25.1)	273 (28.4)	0.165
Men	1107 (72.8)	418 (74.9)	689 (71.6)	0.165
Education				
Illiterate	256 (16.8)	120 (21.5)	136 (14.1)	0.002
Elementary school	586 (38.6)	228 (40.9)	358 (37.2)	0.240
Junior high school	225 (14.8)	77 (13.8)	148 (15.4)	0.497
High school	336 (22.1)	96 (17.2)	240 (24.9)	0.003
College	119 (7.8)	38 (6.8)	81 (8.4)	0.315
Marital status				
Single	54 (3.6)	18 (3.2)	36 (3.7)	0.695
Married	1222 (80.4)	442 (79.2)	780 (81.1)	0.405
Divorced	54 (3.6)	18 (3.2)	36 (3.7)	0.695
Widow or widower	187 (12.3)	80 (14.3)	107 (11.1)	0.121
Mini nutritional assessment				
Normal nutritional status	1082 (71.2)	326 (58.4)	756 (78.6)	0.001
At risk of malnutrition	376 (24.7)	184 (33.0)	192 (20.0)	0.025
Malnourished	61 (4.0)	48 (8.6)	13 (1.4)	0.009
Economic situation, dependent	766 (50.4)	336 (60.2)	430 (44.7)	<0.0001
Smoking	722 (47.5)	267 (47.9)	455 (47.3)	0.809
Drinking	463 (30.5)	164 (29.4)	299 (31.1)	0.503
Regular exercise	617 (40.6)	219 (39.3)	398 (41.4)	0.488
Antihypertensive medication	824 (54.2)	312 (55.9)	512 (53.2)	0.310
Statin	487 (32.0)	201 (36.0)	286 (29.7)	0.011
Hypoglycemic medication	223 (14.7)	108 (19.4)	115 (12.0)	<0.0001
Triple vessel disease	435 (28.6)	228 (40.9)	207 (21.5)	<0.0001
Stents placed	444 (29.2)	198 (35.5)	246 (25.6)	<0.0001
Anemia	356 (23.4)	155 (27.8)	201 (20.9)	0.003
Diabetes mellitus	582 (38.3)	260 (46.6)	322 (33.5)	<0.0001
Hypertension	1092 (71.8)	435 (78.0)	657 (68.3)	<0.0001
Hyperlipidemia	949 (62.4)	364 (65.2)	585 (60.8)	0.088
Cause of admission				
Acute coronary syndrome	654 (43.0)	311 (55.7)	343 (35.7)	<0.0001
Elective PCI	866 (57.0)	247 (44.3)	619 (64.4)	<0.0001

*MACE* major adverse cardiovascular events; *PCI* percutaneous coronary intervention

**Table 2:** Baseline characteristics and biochemical data of the study participants with and without major adverse cardiovascular events

Variable	Total	With MACE	Without MACE	*P-*value
No	1520	558	962	
Age (years)	68.9 ± 12.0	71.9 ± 11.7	67.2 ± 11.9	<0.0001
Body mass index (kg/m^2^)	25.7 ± 10.6	25.2 ± 4.4	26.0 ± 12.9	0.186
Waist circumference (cm)	91.0 ± 10.4	91.9 ± 10.5	90.4 ± 10.2	0.021
Waist to hip ratio	0.94 ± 0.11	0.95 ± 0.14	0.93 ± 0.08	<0.0001
Systolic BP (mmHg)	133 ± 22	133 ± 24	132 ± 21	0.298
Diastolic BP (mmHg)	77 ± 14	75 ± 14	77 ± 13	0.008
HbA1C (%)	7.0 ± 1.8	7.1 ± 1.9	6.9 ± 1.7	0.012
Fasting glucose (mg/dl)	144.9 ± 77.0	159.5 ± 88.7	136.4 ± 68.0	<0.0001
Total cholesterol (mg/dl)	177.1 ± 45.5	174.9 ± 45.3	178.4 ± 45.5	0.156
Triglycerides (mg/dl)	143.8 ± 105.2	138.1 ± 93.3	147.0 ± 111.2	0.116
HDL-cholesterol (mg/dl)	40.2 ± 12.0	38.6 ± 11.9	41.0 ± 12.0	0.0002
LDL-cholesterol (mg/dl)	103.7 ± 35.4	104.4 ± 37.1	103.2 ± 34.3	0.518
Creatinine (mg/dl)	1.74 ± 1.83	2.0 ± 2.3	1.6 ± 1.5	<0.0001
Uric acid (mg/dl)	6.8 ± 2.7	7.0 ± 2.1	6.6 ± 3.0	0.017
eGFR (ml/min/1.73 m^2^)	57.2 ± 23.1	51.4 ± 23.0	60.5 ± 22.6	<0.0001
Hs-CRP (mg/L)	12.2 ± 32.4	14.0 ± 36.8	11.0 ± 29.1	0.001
White blood cell (10^9^/l)	8.469 ± 3.877	8.795 ± 3.699	8.279 ± 3.967	0.012
Red blood cell (10 ^6^/μl)	4.54 ± 1.21	4.46 ± 0.94	4.58 ± 1.34	0.030
Hemoglobin (g/dl)	13.2 ± 2.3	13.1 ± 2.6	13.3 ± 2.1	0.033
Hematocrit (%)	39.5 ± 6.1	39.1 ± 6.3	39.8 ± 5.9	0.039
Mean corpuscular volume (fL)	88.4 ± 7.7	88.6 ± 7.9	88.2 ± 7.6	0.320
MCH (pg/cell)	29.8 ± 7.4	29.6 ± 3.0	30.0 ± 9.1	0.323
MCHC (g/dL)	33.4 ± 1.3	33.3 ± 1.4	33.5 ± 1.2	<0.0001
Malnutrition indicator score	24.7 ± 3.5	23.9 ± 3.6	25.1 ± 3.3	0.010

Data are means ± SD. *MACE* major adverse cardiovascular events, *HDL-C* high-density lipoprotein cholesterol *LDL-C* low-density lipoprotein cholesterol, *Hs-Crp* high-sensitivity C-reactive protein, *MCH* mean corpuscular hemoglobin, *MCHC* mean corpuscular-hemoglobin concentration, *eGFR* = estimated glomerular filtration rate calculated by the chronic kidney disease-EPI two-level race equation [12]

### Baseline Characteristics

All patients received clinical follow-up with a median duration of 23 months (interquartile range: 5–55 months). During the follow-up period, the incidence of MACE was 36.7% (558 of 1,520 patients). The baseline clinical characteristics and data for all of the patients are presented in Table 1. The patients who developed a MACE had higher rates of illiteracy, at risk of malnutrition, malnourished, being economically dependent, taking statins and hypoglycemic medications, and/or having triple vessel disease, stent implantation, anemia, DM, hypertension, and cause of admission for ACS than the patients without a MACE. In addition, the patients who developed a MACE were older and had a higher waist circumference, WHR, HbA1C, fasting glucose, creatinine, uric acid, hs-CRP, white blood cell count, lower diastolic blood pressure, HDL-cholesterol, eGFR, red blood cell count, hemoglobin, hematocrit, mean corpuscular-hemoglobin concentration (MCHC), and malnutrition indicator score than the patients without a MACE (Table 2). Additionally, there was no statistically significant difference in rate of MACE among 4 operators (37.7% vs. 34.1% vs. vs. 39.7% vs. 42.7%, *p* = 0.105).

### Association of the Baseline Clinical and Biochemical Risk Factors for the Development of MACE

After univariate Cox regression analysis, the baseline clinical and biochemical variables associated with MACE were age, being illiterate, a widow or widower, economically dependent, having triple vessel disease, stent implantation, anemia, and DM, WHR, diastolic blood pressure, fasting glucose, total cholesterol, HDL-cholesterol, creatinine, eGFR, red blood cell count, hemoglobin, hematocrit, and MCHC in ACS patients, and age, malnourished, economically dependent, taking hypoglycemic medication, having triple vessel disease, stent implantation, anemia, DM, and hypertension, WHR, fasting glucose, HDL-C, uric acid, creatinine, eGFR, high-sensitivity C-reactive protein, mean corpuscular volume, and MCHC in elective PCI patients. Using multivariate Cox regression analysis, we found the independent factors associated with the development of MACE were triple vessel disease [HR 3.66 (1.38–9.97)], stent implantation [HR 3.61 (1.47–9.05)], hypertension [HR1.73 (1.06–2.21)], and eGFR [HR 0.99 (0.98–1.00)] in ACS patients, and having triple vessel disease [HR 3.12 (1.43–6.82)], stent implantation [HR 4.10 (2.09–8.13)], hypertension [HR 1.07 (1.02–2.37)], and uric acid [HR 1.14 (1.01–1.28)] in elective PCI patients (Tables 3 and 4).

**Table 3:** Cox proportional hazard model of baseline clinical risk factors for the development of major adverse cardiovascular events stratified by acute coronary syndrome and elective PCI subjects

	Acute coronary syndrome				Elective PCI			
Baseline data	Univariate analysis HR (95% CI)	*P* value	Multivariate model HR (95% CI)	*P* value	Univariate analysis HR (95% CI)	*P* value	Multivariate model HR (95% CI)	*P* value
Age	1.02 (1.02–1.03)	<0.0001			1.03 (1.01–1.04)	<0.0001		
Gender	1.10 (0.85–1.45)	0.486			1.15 (0.88–1.54)	0.317		
Education (illiterate)	1.63 (1.19–2.18)	0.002			1.25 (0.85–1.79)	0.257		
Marital status (widow or widower)	1.44 (1.02–1.98)	0.040			0.90 (0.55–1.40)	0.662		
Malnourished	2.87 (0.86–7.21)	0.082			4.00 (1.20–9.91)	0.027		
Economic situation (dependent)	1.52 (1.18–1.97)	0.001			1.81 (1.34–2.45)	0.0001		
Smoking	1.11 (0.89–1.39)	0.369			0.98 (0.76–1.26)	0.857		
Drinking	0.90 (0.68–1.17)	0.414			0.97 (0.71–1.31)	0.849		
Regular exercise	0.85 (0.65–1.11)	0.236			0.79 (0.57–1.07)	0.127		
Antihypertensive medication	0.98 (0.78–1.24)	0.885			0.95 (0.73–1.22)	0.661		
Statins	0.87 (0.69–1.09)	0.222			0.98 (0.74–1.29)	0.909		
Hypoglycemic medication	1.17 (0.89–1.53)	0.262			1.43 (1.01–1.98)	0.042		
Triple vessel disease	1.67 (1.33–2.10)	<0.0001	3.66 (1.38–9.97)	0.009	2.27 (1.75–2.93)	<0.0001	3.12 (1.43–6.82)	0.005
Stent implantation	1.50 (1.18–1.90)	0.001	3.61 (1.47–9.05)	0.005	1.47 (1.12–1.91)	0.005	4.10 (2.09–8.13)	0.0001
Anemia	1.42 (1.10–1.81)	0.007			1.47 (1.09–1.95)	0.011		
Diabetes mellitus	1.78 (1.42–2.23)	<0.0001			1.35 (1.04–1.74)	0.024		
Hypertension	1.27 (0.97–1.67)	0.079	1.73 (1.06–2.21)	0.020	1.50 (1.11–2.07)	0.008	1.07 (1.02–2.37)	0.014
Hyperlipidemia	0.99 (0.78-1.28)	0.960			1.03 (0.79-1.34)	0.839		

*HR* Hazard ratio, *PCI* percutaneous coronary intervention

**Table 4:** Cox proportional hazard model of baseline biochemical risk factors for the development of major adverse cardiovascular events stratified by acute coronary syndrome and elective PCI subjects

	Acute coronary syndrome				Elective PCI			
Baseline data	Univariate analysis HR (95% CI)	*P* value	Multivariate model HR (95% CI)	*P*-value	Univariate analysis HR (95% CI)	*P* value	Multivariate model HR (95% CI)	*P* value
Body mass index	0.98 (0.95–1.01)	0.135			0.99 (0.97–1.03)	0.942		
Waist circumference	1.01 (0.99–1.02)	0.131			1.01 (0.99–1.02)	0.172		
Waist to hip ratio	3.45 (1.30–7.40)	0.016			3.56 (1.29–7.03)	0.019		
Systolic BP	0.99 (0.99–1.00)	0.701			1.01 (0.99–1.01)	0.146		
Diastolic BP	0.24 (0.07–0.90)	0.034			0.15 (0.02–0.97)	0.046		
HbA1C	1.06 (0.99–1.11)	0.063			1.06 (0.99–1.16)	0.192		
Fasting glucose	3.98 (2.24–6.98)	<0.0001			3.82 (1.85–7.66)	0.0004		
Total cholesterol	0.43 (0.24–0.91)	0.030			1.04 (0.30–3.61)	0.946		
Triglyceride	0.99 (0.99–1.00)	0.121			0.99 (0.99–1.00)	0.154		
HDL-cholesterol	0.29 (0.11–0.77)	0.014			0.30 (0.11–0.83)	0.021		
LDL-cholesterol	1.00 (0.99–1.00)	0.447			1.00 (0.99–1.01)	0.221		
Uric acid	1.00 (0.96–1.03)	0.981			1.17 (1.09–1.26)	<0.0001	1.14 (1.01–1.28)	0.041
Creatinine	1.10 (1.03–1.16)	0.005			1.09 (1.05–1.13)	0.0004		
eGFR	0.46 (0.32–0.66)	0.0001	0.20 (0.05–0.86)	0.031	0.32 (0.23–0.48)	<0.0001		
Hs–CRP	1.00 (0.99–1.01)	0.713			1.01 (1.00–1.01)	0.013		
White blood cell	1.02 (0.99–1.05)	0.148			1.01 (0.97–1.05)	0.653		
Red blood cell	0.84 (0.73–0.97)	0.017			0.88 (0.73–1.04)	0.158		
Hemoglobin	0.93 (0.89–0.98)	0.004			0.95 (0.89–1.02)	0.143		
Hematocrit	0.98 (0.96–0.99)	0.003			0.98 (0.95–1.00)	0.073		
MCV	1.00 (0.98–1.01)	0.656			1.02 (1.00–1.04)	0.047		
MCH	0.97 (0.94–1.01)	0.099			1.00 (0.97–1.01)	0.687		
MCHC	0.91 (0.84–0.99)	0.029			0.89 (0.81–0.98)	0.016		

PCI, percutaneous coronary intervention; HR, hazard ratio; HDL-C, high-density lipoprotein cholesterol; LDL-C, low-density lipoprotein cholesterol; MCV, mean corpuscular volume; MCH, mean corpuscular hemoglobin; MCHC, mean corpuscular-hemoglobin concentration. eGFR = estimated glomerular filtration rate calculated by the chronic kidney disease-EPI two-level race equation [12]

### AUC analysis

**Fig. 2:**
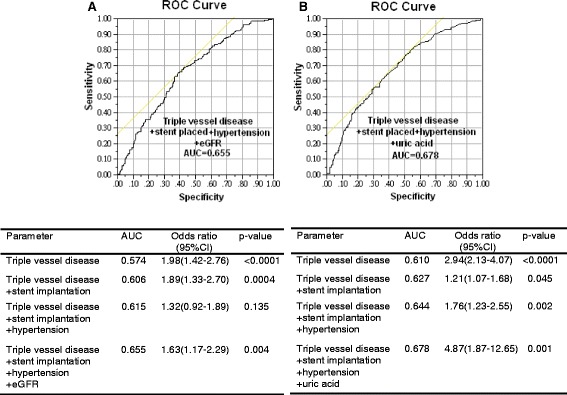
Comparison of the receiver operating characteristic (ROC) curves with area under the curves (AUC) for the risk of major adverse cardiac events (MACE) of acute coronary syndrome subjects with four factors (triple vessel disease, stent implantation, hypertension, and estimated glomerular filtration rate (eGFR)). The AUC using triple vessel disease was calculated first, then stent implantation was added to this model, and then hypertension and eGFR (**a**). In addition, comparison of the ROC curves with area under the curves (AUC) for the risk of MACE of elective percutaneous coronary interventions subjects with four factors (triple vessel disease, stent implantation, hypertension, and uric acid). The AUC using triple vessel disease was calculated first, then stent implantation was added to this model, and then hypertension and uric acid (**b**)

We then evaluated the ability of triple vessel disease, stent implantation, hypertension, and eGFR or uric acid to predict the risk of MACE. In ACS patients, the AUC for triple vessel disease was 0.574 (*p* < 0.0001). When stent implantation, hypertension, and eGFR were added to this multivariate model, the AUC increased to 0.606 (*p* = 0.0004) for triple vessel disease and stent implantation, 0.615 for triple vessel disease, stent implantation, and hypertension, 0.655 (*p* = 0.004) for triple vessel disease, stent implantation, hypertension, and eGFR (Fig. 2a). In elective PCI patients, the AUC for triple vessel disease was 0.610 (*p* < 0.0001). When stent implantation, hypertension, and uric acid were added to this multivariate model, the AUC increased to 0.627 (*p* = 0.045) for triple vessel disease and stent implantation, 0.644 for triple vessel disease, stent implantation, and hypertension, 0.678 (*p* = 0.001) for triple vessel disease, stent implantation, hypertension, and uric acid (Fig. 2b).

### Basic characteristics and biochemical data of the study participants with and without stent implantation

In the present study, we found that stent implantation was associated with the development of MACE. Hence, we further subgrouped our study subjects according to their stent implantation status to examine what reason the stent implantation was associated with MACE. Table 5 lists the basic characteristics and biochemical data of the study participants with and without stent implantation. Patients with stent implantation were younger and had higher frequencies of male gender, triple vessel disease, and hyperlipidemia; higher BMI, HbA1C, total cholesterol, LDL-cholesterol, hs-CRP, and white blood cell count; and lower frequencies of being a widow or widower than those without stent implantation patients.

**Table 5:** Baseline characteristics and biochemical data of the study participants with and without stent implantation

Variable	With stent implantation	Without stent implantation	*P-*value
No	458	1062	
Age (years)	67.2 ± 11.1	69.5 ± 12.3	0.001
Gender (male)	352(76.9)	751(70.7)	0.004
Education (illiterate)	62(13.5)	194(18.3)	0.053
Marital status (widow or widower)	41(9.0)	145(13.7)	0.028
Malnourished	11(2.4)	49(4.6)	0.406
Economic situation (dependent)	215(46.9)	552(52.0)	0.128
Smoking	235(51.3)	490(46.1)	0.077
Triple vessel disease	174(38.0)	254(23.9)	<0.0001
Anemia	103(22.5)	249(23.5)	0.661
Diabetes mellitus	188(41.1)	396(37.3)	0.168
Hypertension	341(74.5)	750(70.6)	0.137
Hyperlipidemia	335(73.1)	613(57.7)	<0.0001
Body mass index (kg/m^2^)	26.7 ± 18.5	25.3 ± 4.4	0.017
Systolic BP (mmHg)	133 ± 21	132 ± 22	0.864
Diastolic BP (mmHg)	77 ± 13	76 ± 14	0.242
HbA1C (%)	7.1 ± 1.7	6.9 ± 1.8	0.048
Fasting glucose (mg/dl)	146.0 ± 75.5	144.3 ± 77.6	0.700
Total cholesterol (mg/dl)	180.7 ± 46.6	175.4 ± 44.7	0.039
Triglycerides (mg/dl)	148.6 ± 108.1	141.3 ± 103.0	0.220
HDL-cholesterol (mg/dl)	39.4 ± 12.7	40.5 ± 11.8	0.110
LDL-cholesterol (mg/dl)	106.7 ± 37.8	102.1 ± 34.0	0.023
Creatinine (mg/dl)	1.9 ± 2.4	1.7 ± 1.5	0.090
Uric acid (mg/dl)	6.9 ± 3.7	6.8 ± 2.2	0.622
eGFR (ml/min/1.73 m^2^)	57.8 ± 22.7	57.3 ± 23.2	0.686
Hs-CRP (mg/L)	13.6 ± 35.5	9.0 ± 24.2	0.044
White blood cell (10^9^/l)	8.652 ± 4.151	8.010 ± 3.128	0.004
Red blood cell (10 ^6^/μl)	4.54 ± 0.85	4.54 ± 1.33	0.952
Hemoglobin (g/dl)	13.3 ± 2.1	13.2 ± 2.3	0.482
Hematocrit (%)	39.7 ± 5.6	39.5 ± 6.2	0.720

Data are means ± SD or number (%). *HDL-C* high-density lipoprotein cholesterol, *LDL-C* low-density lipoprotein cholesterol; *Hs-CRP* high-sensitivity C-reactive protein. eGFR = estimated glomerular filtration rate calculated by the chronic kidney disease-EPI two-level race equation [12]

## Discussion

The goal of this study was to determine the predictors of MACE in Chinese patients with CAD who underwent PCI and/or stenting. Of the 1,520 consecutive patients evaluated, the incidence of MACE was 36.7% after a median duration of 23 months (interquartile range: 5–55 months) of follow-up. Triple vessel disease, stent implantation, hypertension, and eGFR or uric acid all contributed to the risk of a MACE between ACS and elective PCI patients. To the best of our knowledge, this study is the first observational clinical study to identify the independent risk factors for adverse cardiac events in a Chinese cohort of patients, who have distinct differences in presentation from those in western countries [17].

In addition to the most common predictors of MACE in CAD patients such as age [18], female gender [19], hypertension, number of stents placed [19–21], chronic heart failure [20], eGFR [22, 23], anemia [24], HDL- cholesterol [25], and hs-CRP [26], the development of MACE in our population was further determined by triple vessel disease, stent implantation, hypertension, and eGFR or uric acid. Furthermore, we found that triple vessel disease, stent implantation, hypertension, and eGFR predicted the development of MACE in multivariate analysis, with an AUC of 0.655 in ACS patients, and triple vessel disease, stent implantation, hypertension, and uric acid predicted the development of MACE in multivariate analysis, with an AUC of 0.678 in elective PCI patients. In addition, consistent with several previous reports [19, 20] but in contrast to others [26–28], in the present study, DM did not predict the development of a MACE in our patients. Previous studies investigating the impact of DM on clinical outcomes yielded inconsistent results, which likely reflect differences in clinical diagnosis [29–31].

In the current study the levels of eGFR at baseline were inversely associated with the development of MACE in ACS patients, so a low eGFR may have higher risk of MACE, which is consistent with other studies where a lower eGFR was associated with the risk of death, cardiovascular events, and hospitalization [32, 33]. The mechanisms that underlie the association between renal dysfunction and CAD have not been elucidated fully. Previous studies have shown that renal dysfunction is associated with low-grade inflammation and activation of the sympathetic nervous system or the rennin-angiotensinaldosterone system [34–36]. Foley et al. demonstrated that calcium-phosphate production and oxidative stress were shown to promote renal dysfunction [37]. Muntner et al. showed that apolipoprotein A1 levels are decreased and levels of homocysteine, lipoprotein (a), fibrinogen, and CRP are increased among patients with CKD [38]. These factors above could also contribute to the pathogenesis of atherosclerosis. Herzog and colleagues also described pressure overload, volume overload, and CKD associated non-hemodynamic factors that have enhanced CKD to alter the myocardium [39]. Renal insufficiency is an independent risk factor for cardiovascular outcomes and in-hospital and late mortality [40, 41]. Increased uremic toxins such as total indoxyl sulfate and *p*-cresylsulphate may be involved in the pathogenesis of coronary atherosclerosis, prolonged QTc interval, left ventricular systolic dysfunction, and elevated risk of MACE in CAD patients with low estimated glomerular filtration rate, and it is considered a coronary heart disease risk equivalent [42–46]. In addition, we found that uric acid was an independent risk factor for developing a MACE in elective PCI patients. Previous clinical investigations have demonstrated that hyperuricemia is an independent predictor of mortality in patients with cardiovascular disease [47–49]. Uric acid is the final oxidation product of purine catabolism in humans. It is formed from the breakdown of adenosine and guanine. When the purine breakdown products xanthine and hypoxanthine are converted to uric acid through the enzymatic action of xanthine oxidase, molecular oxygen is reduced and the free radical superoxide is generated. Oxygen-free radicals are postulated to play a key role in vascular injury in cardiovascular and kidney disease. George et al. suggested that uric acid was harm to endothelial function leading to cause of cardiovascular disease because uric acid levels may act as a marker of xanthine oxidase activity [50]. Previous studies have also shown that uric acid has directly contributed proliferative, proinflammatory, and proatherogenic effects on human vascular smooth muscle cells and endothelial cells through upregulate CRP, an active component in the inflammatory process, which can lead to both hypertension and vascular disease [51–53]. Taken together, these findings highlight the clinical and public health importance of chronic renal insufficiency and hyperuricemia.

In this study, we found that hypertension was an independent risk factor for developing a MACE not only in ACS patients but also in elective PCI patients. This is consistent with reports in which a history of hypertension was one of the risk factors for a MACE [21, 54]. Madan et al. enrolled 9,494 patients and found that hypertension was an independent predictor of adverse cardiac events after PCI [20]. In the other hand, chronic hypertension is also one of the important cardiovascular risk factors for development of atherosclerosis [55] and an increased incidence of CAD [56], CKD [57], peripheral vascular disease [58], and cerebrovascular disease [59]. Moreover, hypertension is also known as an important risk factor for myocardial infarction, stroke, heart failure, and cardiovascular death [60, 61]. The relation between hypertension and myocardial infarction could be explained underscoring two key factors: (1) common risk factors, such as sympathetic hyperactivity, insulin resistance, vasoactive substances (i.e., angiotensin II), and genetic risk profiles and (2) hypertension is a risk factor of atherosclerosis, which contributes to progression of myocardial infarction [62]. Genetic risk factors such as gene polymorphisms of the angiotensinogen-converting enzyme and of the renin-angiotensin-aldosterone system, could cause myocardial infarction, hypertension, and cardiovascular complications [63, 64]. Furthermore, in hypertensive patients, a hyperactivity of sympathetic tone which causes sympathetic vasoconstriction on glucose extraction in skeletal muscle, beta-adrenergic receptor-mediated insulin resistance, and vascular rarefaction; may contribute atherosclerosis by worsening insulin resistance. Previous study suggested that sympathetic hyperactivity itself also contributes higher risk of coronary spasm, coronary thrombosis, and sudden death [65].

Besides the classical risk factors, others risk factors may also influence the development of MACE [20]. In the present study, we also found that triple vessel disease and stent implantation were associated with the development of MACE. Studies by Chow et al. and de Waha et al. both reported that the severity of CAD and multivessel CAD not only predicted all-cause mortality but also were high-risk factors for adverse clinical outcomes [66, 67]. The higher morbidity and mortality are seen in ST-segment elevation myocardial infarction patients with multi-vessel CAD. The mechanism is multifactorial, including total ischemic burden, the presence of diffuse atherosclerosis as a harbinger of plaque instability, and impaired contractility of non-infarct zones in the presence of multiple obstructive stenosis [68]. Interestingly, in our study, the stent implantation was found to be a surrogate marker of MACE. When we further subgrouped our study subjects according to their stent implantation status, the results found that patients with stent implantation had higher rates of triple vessel disease and hyperlipidemia; higher BMI, HbA1C, total cholesterol, LDL-cholesterol, hs-CRP, and white blood cell count than those without stent implantation patients. Hyperlipidemia and obesity are recognized as a risk factor for CAD and coronary mortality [69, 70]. Furthermore, several reports have suggested that delayed arterial healing and vessel remodeling due to chronic inflammation have been reported as reasons for the high rates of very late stent thrombosis with drug-eluting stent [71–75]. Previously, studies have indicated that this reduction in restenosis might have been obtained at the expense of a higher incidence of stent thrombosis that links stent implantation and adverse cardiac events such as recurrent myocardial infarction or death [76, 77]. Hence, we think multifactorial occurrence, including triple vessel disease, hyperlipidemia, higher BMI, HbA1C, and inflammation response that promote the association of stent implantation with the MACE in our study [66, 67, 69, 70, 75, 78]. In addition, there was no statistically significant difference in rate of MACE among 4 operators in the present study. This indicated that operators issue did not affect the finding of stent implantation association with MACE.

There are several limitations to this study. First, the results may be skewed by procedural preferences and the patients seen at our institution. Second, we did not assess several complications that are common after PCI such as contrast nephropathy and access-site vascular complications. Third, we did not examine the relationship between operator procedure volume and cardiac complications. Four, this is an observational study in only one centre. Hence, further work is required to confirm these findings in populations of different races and these exploratory results should be confirmed in a larger study and other centre. Finally, although the current model has been validated for the contemporary practice of PCI, further advances in PCI techniques and technology may reduce the discriminatory ability and predictive accuracy of the model. Thus, the model may need to be recalibrated in the future.

## Conclusion

The present study indicates that having triple vessel disease, stent implantation, hypertension, and eGFR or uric acid levels independently predicted the development of MACE in patients with CAD after long-term follow-up. We suggest that uric acid, eGFR, and blood pressure should be closely monitored in patients with CAD.

## Abbreviations

ACS: acute coronary syndrome
AUC: area under the curves
BMI: body mass index.
CABG: coronary artery bypass graft
CAD: Coronary artery disease
CKD: Chronic kidney disease
eGFR: Estimated glomerular filtration rate
HbA1c: Hemoglobin A1c
HDL: High-density lipoprotein
HF: Heart failure
CRP: C-reactive protein
HR: Hazard ratio
LDL: Low-density lipoprotein
MACE: Major adverse cardiac events
MCHC: Mean corpuscular-hemoglobin concentration
MI: Myocardial infarction
PCI: Percutaneous coronary intervention
RBC: Red blood cell
ROC: Receiver operating characteristic
WBC: White blood cell
WHR: Waist to hip ratio
